# Negative back beliefs are associated with increased odds of low back pain and disability: a 10-year cohort study in men

**DOI:** 10.1093/rheumatology/kead587

**Published:** 2023-11-08

**Authors:** Mahnuma M Estee, YuanYuan Wang, Stephane Heritier, Donna M Urquhart, Flavia M Cicuttini, Mark A Kotowicz, Sharon L Brennan-Olsen, Julie A Pasco, Anita E Wluka

**Affiliations:** School of Public Health and Preventive Medicine, Monash University, Melbourne, VIC, Australia; School of Public Health and Preventive Medicine, Monash University, Melbourne, VIC, Australia; School of Public Health and Preventive Medicine, Monash University, Melbourne, VIC, Australia; School of Public Health and Preventive Medicine, Monash University, Melbourne, VIC, Australia; School of Public Health and Preventive Medicine, Monash University, Melbourne, VIC, Australia; Deakin University, IMPACT—Institute for Mental and Physical Health and Clinical Translation, Geelong, VIC, Australia; Department of Medicine–Western Health, The University of Melbourne, St Albans, VIC, Australia; University Hospital Geelong, Barwon Health, Geelong, VIC, Australia; Australian Institute for Musculoskeletal Sciences (AIMSS), Western Health and University of Melbourne, St Albans, VIC, Australia; School of Health and Social Development, Deakin University, Geelong, VIC, Australia; School of Public Health and Preventive Medicine, Monash University, Melbourne, VIC, Australia; Deakin University, IMPACT—Institute for Mental and Physical Health and Clinical Translation, Geelong, VIC, Australia; Department of Medicine–Western Health, The University of Melbourne, St Albans, VIC, Australia; University Hospital Geelong, Barwon Health, Geelong, VIC, Australia; School of Public Health and Preventive Medicine, Monash University, Melbourne, VIC, Australia

**Keywords:** low back pain, disability, back beliefs, cohort study

## Abstract

**Objective:**

Although negative back beliefs are associated with high-intensity low back pain (LBP)/disability, whether they influence incident high-intensity LBP/high disability over the long-term is unknown. This study aimed to investigate whether negative back beliefs were associated with developing high-intensity LBP and/or high disability over 10 years in men.

**Methods:**

Men with no or low-intensity LBP and/or disability attending the Geelong Osteoporosis Study between 2006 and 2010 were included. Data on age, body mass index, mobility, education, back beliefs (Back Beliefs Questionnaire), LBP and disability (Graded Chronic Pain Scale) were collected between 2006 and 2010. Beliefs, LBP and disability were re-assessed in 2016–2021. Binary logistic regression was used to examine the association between negative back beliefs and incident high-intensity pain and/or high disability, adjusting for age, body mass index, mobility and education.

**Results:**

At baseline, 705 participants (mean age 53.8 years) had no or low LBP and no or low disability; 441 (62.6%) participants completed a 10-year follow-up. Of these, 37 (8.4%) developed high-intensity pain and/or high disability. In multivariate analyses, participants with more negative back beliefs at baseline were more likely to develop high-intensity pain and/or high disability (odds ratio 1.05; 95% CI: 1.00, 1.11). Developing more negative back beliefs was also associated with incident high-intensity pain and/or high disability (odds ratio 1.20; 95% CI: 1.12, 1.30).

**Conclusion:**

In a male community-based population, negative beliefs regarding the consequences of LBP were associated with an increased likelihood of developing high-intensity pain and/or high disability. Addressing negative back beliefs in the community may reduce the incidence of high-intensity pain and/or high disability over 10 years in men.

Rheumatology key messages

- Holding negative beliefs about back pain-related consequences increases likelihood of developing high-impact LBP in men.
- Addressing negative beliefs in people with low-intensity LBP may reduce the transition to high-impact LBP.

## Introduction

Chronic low back pain (LBP), the leading cause of global disability, reduces health-related quality of life and has a lifetime prevalence of ≥85% [1]. In 2019, LBP affected ∼568 million people, resulting in 65 million LBP-related years lived with disability [1]. In the UK, LBP was estimated to be responsible for 12.5% of all work absences [2]. It is the condition with the highest healthcare cost in the USA [3]. The recommended treatments for chronic LBP have limited efficacy [4], with 43% of people with LBP dissatisfied with their medical care [5]. Lack of generalizability of clinical trial results to community populations, comorbidities and the provision of care that is non-adherent to guidelines may explain the lack of efficacy and dissatisfaction with LBP treatments [6]. It is critical to target modifiable factors for prevention.

LBP tends to fluctuate throughout life between pain-free periods and episodes of minor to moderate pain/disability [7]. However, a small portion of people develop high-intensity LBP, which is more likely to be persistent [7]. These individuals are more likely to use multidisciplinary care and health-related expenses and be affected by poor quality of life, reduced work productivity and impaired daily activities [8, 9]. Therefore, identifying factors associated with the transition to high-intensity pain and/or high disability is important in order to avoid this occurring.

Community back pain-related beliefs regarding consequences have been associated with poor outcomes. Cross-sectional studies have demonstrated an association between negative back beliefs and severe LBP and disability [10]. However, there are only a few cohort studies examining the relationship between patients' back beliefs and incident LBP and disability, with limited literature largely related to women, over 1–2 years [11–13]. As health-related beliefs/behaviours and pain perception differ according to sex and the prevalence of back problems is higher in working-age men with greater economic impact, the relationship between back beliefs and LBP outcomes should be examined separately in women and men [14–17]. No study has examined the longitudinal association of negative back beliefs with developing high-intensity pain and/or high disability in community-based men over the long term. As negative beliefs relating to back pain are potentially modifiable [18], whether back beliefs affect the incidence of LBP and disability in men deserves attention.

Therefore, this study aimed to examine whether in Australian men without high-intensity LBP and high disability, holding negative back beliefs was associated with an increased incidence of high-intensity pain and/or high disability 10 years later.

## Method

### Study population

**Figure 1.**
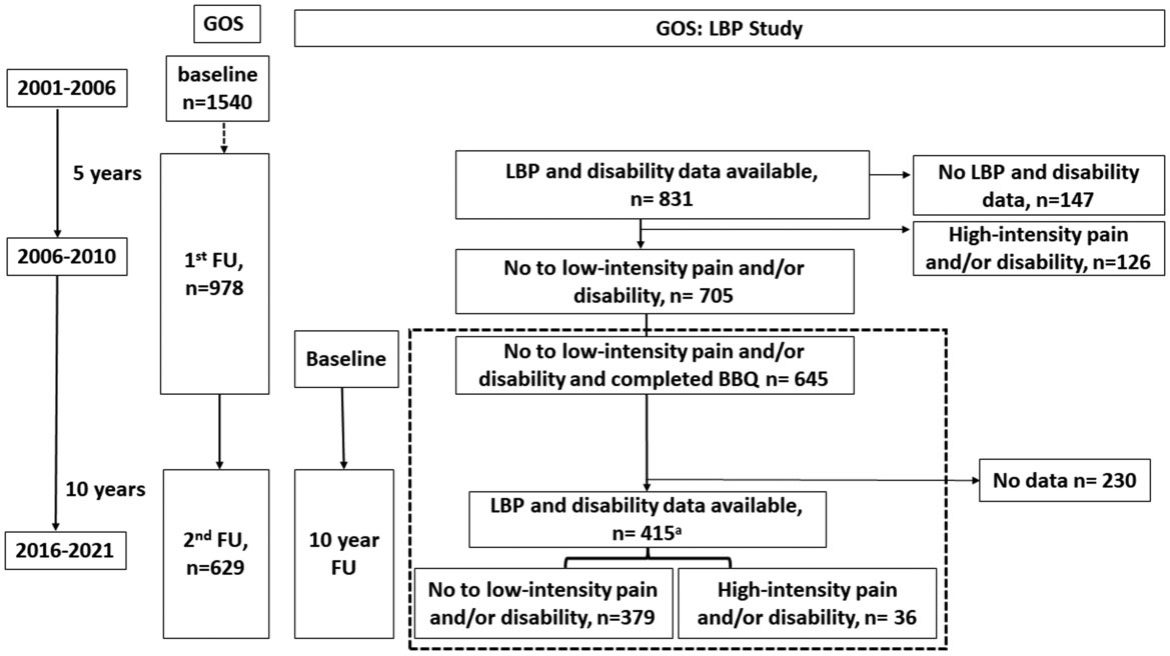
Flow chart showing the number of participants in the main Geelong Osteoporosis Study (GOS) study and current low back pain (LBP) study within the GOS. The dotted box indicates the current study timeline between baseline (2006–2010) to follow-up (2016–2021). ^a^The number of participants providing LBP data was 417, and 32 participants developed high-intensity pain after 10 years; 415 participants provided disability data and 14 participants developed high disability after 10 years. BBQ: Back Belief Questionnaire; FU: follow-up

The Geelong Osteoporosis Study (GOS) is a population-based prospective cohort study of Australian adults. The men's study recruited 1540 men aged ≥20 years from the Barwon Statistical Division using the Australian electoral roll between 2001 and 2006 (Fig. 1) [19]. This sample is representative of the Australian population and all participants gave written informed consent [19]. Follow-up at 5 years involved 978 men (2006–2010). There were no statistical differences in age, mood disorder, education and mobility between those presenting at 5 years and those who did not [20]. At 15 years (2016–2021), 629 men returned for follow-up. The current cohort study was nested within the GOS, with inception at the 5-year follow-up (referred to as baseline) (Fig. 1, dotted box), including participants providing LBP and disability data at both 2006–2010 and 2016–2021 (referred to as follow-up).

### Low back pain and disability

The Graded Chronic Pain Scale (GCPS) was used to collect information regarding LBP and disability in the past 6 months [21]. Participants were categorized into five groups according to the original GCPS (combination of pain-intensity score and disability point scores) [21]. Participants were further categorized into two groups: having no or low-intensity pain and no or low disability (grade 0 or 1), and high-intensity pain and/or high disability (grade 2, 3 or 4), at baseline and 10 years’ follow-up. This study included participants having no or low-intensity pain and no or low disability at baseline. Those with grade 2, 3 or 4 at follow-up were categorized as developing high-intensity pain and/or high disability (referred to as high symptoms group) and the rest were categorized as having no or low-intensity pain and/or disability (referred to as no or low symptoms group). Participants were also categorized according to pain (no pain or low-intensity pain if pain intensity score between 0 and < 50, and high-intensity pain if pain intensity score ≥50), and according to disability status (no or low disability if disability points <3 and high disability if disability points ≥3) [21].

### Back beliefs

Individuals' general beliefs about LBP and its inevitable consequences were captured using the self-administered Back Belief Questionnaire (BBQ) [22]. BBQ comprises 14 statements, each with a five-point Likert scale, where 1 = completely disagree and 5 = completely agree. Out of the 14 statements, nine were included to evaluate back beliefs whereas five were distractors. The individual score is reversed to calculate total scores ranging from 9 to 45, with lower scores indicating more negative beliefs.

### Demographic data and education

In 2006–2010, age was recorded, and weight without bulky clothes and height without shoes were measured. BMI was calculated. Participants self-reported their highest completed education level, which was categorized into not completed secondary school *vs* completed secondary school [20].

### Depression and mobility

Depressive features were captured using the self-reported Hospital Anxiety and Depression Scale (HADS-D) questionnaire (score 0–21); higher score indicates depressive symptoms [23]. Mobility status was assessed by asking ‘How would you best describe your activity now?’, with participants categorized as high mobility or low mobility [20].

### Statistical analysis

The characteristics of participants with no or low symptoms were compared with those with high symptoms using an independent samples Student’s *t*-test and χ^2^ test. The mean difference (MD) was calculated. Binary logistic regression was used to assess the associations between back beliefs and the odds of developing high-intensity pain/high disability, adjusted for age, BMI, mobility and education. Depressive symptoms was not included in the regression model as it is potentially on the causal pathway between negative back beliefs and developing LBP [24]. The association was also evaluated with developing any pain and/or disability or developing high-intensity pain and/or disability from low symptoms.

Change in back beliefs was calculated by subtracting baseline back belief score from follow-up score, and thus a negative score indicates the development of more negative beliefs. Binary logistic regression was used to assess the associations between change in back beliefs and the odds of developing of high-intensity pain/high disability, adjusted for age, BMI, mobility, education and baseline back belief scores.

Exploratory subgroup analyses were performed based on depression, BMI, age, mobility and education and were not adjusted for multiple testing. For subgroup analysis, age and BMI were categorized based on median values, depression was categorized (HADS-D ≥ 8, indicating depressive features), mobility was categorized as high or low mobility and education was categorized as not completed secondary school *vs* completed secondary school. Interaction between groups was assessed [23].

We examined the prevalence of negative beliefs for each statement in those with no or low-intensity pain and/or disability and in those with high-intensity pain and/or high disability. For each statement, we categorized the response into negative back belief (if responded 4 or 5) *vs* positive belief or none (if responded 1, 2 or 3) [25–27]. The association between a negative response for each statement with developing high-intensity pain and/or high disability was evaluated. We then calculated the number needed to be exposed (NNE) to each negative back belief using the adjusted odds ratio (OR) that estimates the number of people needed to be exposed to a particular negative back belief to develop one extra case of high-intensity pain and/or high disability [28]. A *P*-value <0.05 was considered statistically significant. All statistical analyses were performed using IBM SPSS Statistics (v 27.0; IBM Corp., Armonk, NY, USA).

### Ethics

This study was approved by the Human Research Ethics Committees of Barwon Health (Reference Number 0056_E7), and a research collaboration agreement was approved between Monash University and Barwon Health.

## Results

In 2006–2010, 705 participants had no or low-intensity pain and no or low disability, with 645 (91.5%) participants providing back beliefs data. The number of participants providing pain and disability data in 2016–2021 was 415 (64.3%) (Fig. 1). Pain but not disability data were available for 417 participants. Participants completing the study were younger, less depressed, held more positive back beliefs and had higher mobility than those who did not complete (Supplementary Table S1, available at *Rheumatology* online). In 2006–2010, 645 (91.5%) participants provided back beliefs data. In 2016–2021, 36 (8.6%) had high-intensity pain and/or high disability, 32 (7.7%) had high-intensity pain, and 14 (3.4%) had high disability.

Men with no or low symptoms were compared with men with high symptoms (Table 1 and Supplementary Table S2, available at *Rheumatology* online). Compared with men with no or low symptoms, those who developed high-intensity pain and/or high disability were more depressed (MD −1.4; 95% CI: −2.4, −0.4), held more negative beliefs (MD 2.6; 95% CI: 0.4, 4.90) and had lower mobility (33.3% *vs* 17.7%); those developing high-intensity pain were more depressed (MD −1.38; 95% CI: −2.2, 0.6) and had lower mobility (34.4% *vs* 17.7%); and those developing high disability were more depressed (MD −2.2; 95% CI: −3.9, −0.5), and held more negative beliefs (MD 3.8; 95 CI: 0.4, 7.3). There were no differences in age, BMI and educational level between the groups. Back beliefs became more negative in those who developed high-intensity pain (MD 4.7; 95% CI: 2.2, 7.2) or high disability (MD 6.0; 95% CI: 2.4, 9.7) or both high-intensity pain and/or high disability (MD 4.7; 95% CI: 2.3, 7.0).

**Table 1.** Baseline characteristics of study participants

**Variable**	**Pain and/or disability** ^a^			**Pain** ^b^			**Disability** ^a^		
	No or low-intensity pain and/or disability	High-intensity pain and/or disability	*P*	No or low-intensity pain	High -intensity pain	*P*	No or low disability	High disability	*P*
	(*n* = 379)	(*n* = 36)		(*n* = 385)	(*n* = 32)		(*n* = 401)	(*n* = 14)	
Baseline data									
Age^c^, mean (s.d.), years	53.3 (13.9)	55.7 (13.6)	0.32	53.5 (13.9)	55.6 (13.6)	0.39	53.5 (14)	55.9 (10.9)	0.53
BMI^c^, mean (s.d.), kg/m^2^	27.3 (3.9)	26.7 (4.2)	0.37	27.3 (3.9)	26.9 (4.4)	0.65	27.2 (3.9)	27.2 (4.8)	0.99
Depressive features^c^	2.4 (2.2)	3.8 (2.9)	0.01	2.4 (2.2)	3.8 (3.0)	0.02	2.4 (2.2)	4.6 (2.9)	0.02
Not completed secondary school^d^, *n* (%)	148 (39.8)	15 (41.7)	0.83	150 (39.7)	15 (46.9)	0.43	158 (40.1)	5 (35.7)	0.74
Low mobility^d^, *n* (%)	67 (17.7)	12 (33.3)	0.02	68 (17.7)	11 (34.4)	0.02	74 (18.5)	5 (35.7)	0.11
Back belief^c^^,^^e^, mean (s.d.)	30.6 (6.5)	28 (6.1)	0.02	30.6 (6.5)	28.4 (6.3)	0.06	30.5 (6.5)	26.7 (5.6)	0.03
Change in score over 10 years									
Back belief score^c^^,^^f^, mean (s.d.)	1.7 (6.9)	−3.0 (6.1)	<0.001	1.6 (6.9)	−3.1 (6.4)	<0.001	1.4 (6.9)	−4.6 (1.1)	0.001

Baseline characteristics of study participants comparing those who had persistent no or low-intensity pain and no or low disability and those who developed high-intensity pain and/or high disability at 10 years follow-up.

a Data available for 441 participants who provided pain, disability and belief data at both time points.

b Data available for 443 for participants who provided pain and back belief, but not disability data at both time points.

c Comparison *P*-value for independent *t*-test.

d Comparison *P*-value for χ^2^ test.

e Lower back belief score indicates more negative back beliefs.

f Change in back beliefs was calculated by subtracting baseline (2006–2010) back belief score from follow-up (2016–2021), a negative value indicates developing more negative belief scores.

Men with no-disability at baseline but who developed any disability at 10 years had lower back belief scores than those who did not develop any disability (MD 3; 95% CI: 0.8, 5.1) (Supplementary Table S3, available at *Rheumatology* online). Men with low-intensity pain and/or disability at baseline who deteriorated over 10 years had lower back belief scores than men who improved or had persistent low-intensity pain and/or disability at 10 years (MD 2.8; 95% CI: 0.2, 5.3) (Supplementary Table S3, available at *Rheumatology* online).

### Back beliefs and developing high-intensity pain and/or high disability

The associations between age, BMI, depression, mobility, education and back beliefs with developing high-intensity pain and/or high disability were examined (Table 2). Low mobility was associated with increased odds of developing high-intensity pain and/or high disability and high-intensity pain.

**Table 2.** Associations between negative back beliefs and developing high-intensity pain and/or high disability

	OR (95% CI)					
	Pain and/or disability		Pain		Disability	
	Unadjusted	Adjusted	Unadjusted	Adjusted	Unadjusted	Adjusted
Negative back beliefs at baseline						
Age	1.01 (0.99, 1.04)	—	1.01 (0.99, 1.04)	—	1.01 (0.97, 1.05)	—
BMI	0.96 (0.88, 1.05)	—	0.98 (0.89, 1.08)	—	1.0 (0.87, 1.14)	—
Education	1.08 (0.54, 2.17)	—	1.34 (0.65, 2.77)	—	0.83 (0.27, 2.52)	—
Mobility	2.32 (1.11, 4.89)	—	2.44 (1.13, 5.30)	—	2.46 (0.80, 7.54)	—
Negative back beliefs	1.06 (1.01, 1.12)	1.05 (1.00, 1.11)^a^	1.05 (1.00, 1.11)	1.04 (0.99, 1.10)^a^	1.09 (1.01, 1.18)	1.08 (1.00, 1.17)^a^
Change in back beliefs						
Developing more negative back beliefs	1.11 (1.05, 1.16)	1.20 (1.12, 1.30)^b^	1.11 (1.04, 1.16)	1.19 (1.11, 1.27)^b^	1.14 (1.05, 1.22)	1.32 (1.15, 1.49)^b^

a Adjusted for age, BMI, mobility (low mobility) and education (completed secondary school or lower).

b Adjusted for age, BMI, mobility, education and baseline back belief score. OR: odds ratio.

Negative back beliefs were associated with increased odds of developing high-intensity pain and/or high disability (OR 1.06; 95% CI: 1.01, 1.12) and high disability (OR 1.09; 95% CI: 1.01, 1.18), in univariable analyses. These associations persisted in multivariable analyses (respectively OR 1.05; 95% CI: 1.00, 1.11; and OR 1.08; 95% CI: 1.00, 1.17) after adjustment for age, BMI, mobility and education.

Subgroup analyses in men with no symptoms and men with low symptoms were performed (Supplementary Tables S3 and Table S4, available at *Rheumatology* online). Low back beliefs scores were significantly associated with increased odds of developing any disability in men with no disability at baseline, in adjusted analysis (OR 1.06; 95% CI: 1.02, 1.12) but not in those with low pain and/or disability. Men with low-intensity pain and/or disability at baseline were not at increased odds of developing high-intensity pain and/or high disability at 10 years in adjusted analyses (OR 1.04; 95% CI: 0.98, 1.10).

The relationship between developing more negative back beliefs over 10 years with incident high-intensity pain and/or high disability was examined. Developing more negative back beliefs was associated with increased odds of high intensity-pain and/or disability (OR 1.20; 95% CI: 1.12, 1.30), high-intensity pain (OR 1.19; 95% CI: 1.11, 1.27) and high disability (OR 1.32; 95% CI: 1.15, 1.49) after adjustment for age, BMI, mobility, education and baseline back beliefs scores (Table 2).

We performed exploratory subgroup analyses to determine whether the results were related to depression, BMI, age, mobility and education. There were no significant interactions between groups (Supplementary Table S5, available at *Rheumatology* online).

The associations between individual negative beliefs and incident high-intensity pain and/or high disability were also examined for significant OR (Supplementary Table S6, available at *Rheumatology* online). The 18.3% of men holding the negative belief ‘Back trouble will eventually stop you from working’ were at increased odds of developing high-intensity pain and/or high disability (OR 2.69; 95% CI: 1.23, 5.76) and high-intensity pain (OR 2.81; 95% CI: 1.27, 6.19). The NNE for holding this belief was 5, indicating that for every five men holding this negative belief, an additional man developed high-intensity pain and/or disability or developed high-intensity pain. The negative belief that ‘Back trouble must be rested’, held by 25% of the population, was associated with developing high disability (OR 3.74; 95% CI: 1.26, 11.1): the NNE for holding this negative belief was 3. This signifies that to develop one extra new case with high disability, three people need to hold this negative belief.

## Discussion

This study is the first population-based cohort study examining the relationship between back beliefs and incident high-intensity pain and/or high disability in men followed for at least 10 years. It showed that men who held negative back beliefs at baseline were more likely to develop high-intensity pain and/or high disability 10 years later than those who held more positive back beliefs.

There have been only three longitudinal studies, performed over 1–2 years, relating baseline back beliefs to incident LBP [11–13]. These showed inconsistent results but none examined the relationship between developing high-intensity LBP and/or high disability as was examined in the current study. All three previous studies examined pain as an outcome: Elfering *et al.* identified any incident pain as the outcome, but Ng *et al.* and Alyousef *et al.* examined high-intensity pain as in the current study [11–13]. Our results are consistent with those of Elfering and Alyousef, showing no relationship between negative back beliefs and incident pain [11, 12]. In contrast, Ng showed increased odds of developing pain, but this study was performed in a population that was obese (mean BMI 32.4 kg/m^2^) and largely female (76%) [13]. The only previous study to consider disability, by Alyousef, reported no increased odds related to negative back beliefs, but the population differed from the current study in that it consisted only of women, and a different definition of high disability was used [11]. This may be explained by known gender differences in pain perception (higher clinical pain, pain sensitivity but lower pain inhibition in women), beliefs about health (more men report good health) or repetitive negative thinking (more in women) [14, 16, 17]. Thus, this is the first longitudinal study to consider high-impact LBP (considering both pain and disability) in men over 10 years.

Our study has shown, as previous studies, that back beliefs may change over time [29, 30]. We observed an association between developing more negative back beliefs and incident high-impact back symptoms. However, this relationship may be bidirectional, as those with high-intensity low back pain and/or disability are more likely to hold more negative back beliefs [25, 26, 31]. As our study took place over 10 years, and we do not have information about back beliefs and LBP in the interim, it is not possible to determine whether incident high-intensity back symptoms preceded the change in beliefs or vice versa. However, it is known that negative beliefs may alter behaviour, affecting depression and mobility and thus impacting incident pain and/or disability [32–34]. Beliefs and behaviour may be affected by initial and early interactions with health care providers: this may impact long-term healthcare outcomes [35]. Educational programs and media campaigns have been shown to improve patients’ and community back beliefs [29, 30]. Improvement in community back beliefs has been shown to reduce the burden of LBP [29]. Therefore, it is possible that attention during the early contact with health care providers for LBP to address negative back beliefs may have long-term consequences, reducing the incidence of high-intensity pain and/or high disability. Our data suggest that some beliefs may be more important that others such as ‘Back trouble will eventually stop you from working’ and ‘Back trouble must be rested’. Whether addressing these specific beliefs will reduce the burden related to high-intensity pain and/or high disability needs to be tested.

The strengths of the current study include that it is the only prospective cohort study with over 10 years’ follow-up to examine the relationship between back beliefs and LBP outcomes, in a representative community-based cohort of men. For the first time, the relationship between negative back beliefs and high impact back symptoms, which account for much of the burden of LBP, was examined. Given the high burden of LBP in working age men, it is important to examine this in men with no and low-intensity pain and disability, in order to prevent the transition of low-intensity LBP to high-intensity pain and disability [15]. Validated self-reported questionnaires were used to collect data on LBP and beliefs [21, 22].

Nevertheless, this study has a number of limitations including that the follow-up rate was 64.3%. This may have introduced some selection bias. Those who were lost to follow-up were older, more depressed, held more negative beliefs, did not complete secondary school and were less mobile than those who completed the study. Thus, our results may have underestimated the relationship between negative back beliefs and incident high-intensity back pain and/or disability and be more generalizable to a healthier community-based male population. However, subgroup analyses did not suggest that the observed relationships differed in participants who were older, depressed, with high BMI, did not complete secondary school and had low mobility, suggesting results may be generalizable to a community-based male population. It is possible that there may be outcome misclassification as back symptoms were assessed at two time points over 10 years. However, although LBP tends to be episodic when it is of low to moderate severity, once high-intensity LBP or disability develops, symptoms tend to be persistent [7]. We have used high-intensity LBP and/or disability as the outcome measure to avoid this misclassification: we demonstrated significant relationships. Where non-differential misclassification is present, the results tend to underestimate relationships. Although we do not have clinical information regarding the cause of LBP in study participants, almost 90% of LBP is known to be non-specific, with no specific cause identified [36, 37]. While it is possible that medication used during the course of the study may have influenced the trajectory of LBP if present, data regarding medication use during the study period was not available. Although occupational factors have been shown to influence incident LBP, information regarding relevant occupational exposures for LBP was not available at study inception to enable the inclusion of occupational factors in the analysis.

## Conclusion

In a male, largely healthy, community-based population with no or low-intensity back pain and/or disability, participants holding negative beliefs about LBP-related consequences were more likely to develop high-intensity pain and/or high disability over 10 years. These results may be less generalizable to older, less healthy and more depressed men. Attention to address and correct negative back beliefs at an early stage in the course of LBP may be important in reducing the transition of low-intensity pain/disability to high-intensity pain and/or high disability over the long-term.

## Supplementary Material

kead587_Supplementary_Data

## Data Availability

The data underlying this article may be shared on reasonable request to the corresponding author.
