# Supplementary material for: Negative back beliefs are associated with increased odds of low back pain and disability: a 10-year cohort study in men
Source: Rheumatology (Oxford). 2023 Nov 8;63(12):3353–9. doi: 10.1093/rheumatology/kead587 (PMC11636553; doi:10.1093/rheumatology/kead587)
Supplement: kead587_Supplementary_Data [file kead587_supplementary_data.docx]

**Supplementary Table S1: Difference between those who completed follow-up in the Back-pain study (continuing population) and those who did not (loss to follow-up, LTFU)**

|  | Study population^1^  n=415 | Lost to follow up^2^  n= 230 | Significance for difference |
| --- | --- | --- | --- |
| Age^3^, years | 53.5 (13.9) | 65.5 (18.7) | <0.001 |
| BMI^3^, kg/m^2^ | 27.2 (4) | 27.2 (4.2) | 0.87 |
| Depressive features^3^ | 2.5 (2.3) | 3.2 (2.3) | <0.001 |
| Not Completed secondary school^4^ | 163 (40%) | 112 (49.1%) | 0.03 |
| Low mobility^4^ | 79 (19%) | 70 (30.6%) | <0.001 |
| Back belief^3^, (total score) | 30.4 (6.5) | 28.9 (6.6) | 0.004 |

^1^Study population: participants who completed the chronic pain grade questionnaire at baseline (2006-2010) and follow-up (2016-2021)

^2^ LTFU: Participants who completed the chronic pain grade questionnaire at baseline (2006-2010) but not at follow-up (2016-2021)

^3^Data presented as mean (standard deviation); comparison p-value for Independent T-test

^4^Data presented as number (percentage); comparison p value for Chi-square Test

**Supplementary Table S2: Characteristics of study participants, in those who had persistent no or low-intensity pain and no or low disability and those who developed high-intensity pain and/or high-disability**

| **Variables** | **Pain and/or disability^1^** | | | **Pain^2^** | | | **Disability^1^** | | |
| --- | --- | --- | --- | --- | --- | --- | --- | --- | --- |
|  | No or low-intensity pain and/or disability  n = 379 | High-intensity pain and/or disability  n = 36 | P | No or low-intensity pain  n = 385 | High-intensity pain  n = 32 | P | No or low-disability  n = 401 | High-disability  n = 14 | P |
| Age^3^,  years | -2.4  (-7.2, 2.4) | | 0.32 | -2.2  (-7.2, 2.8) | | 0.39 | -2.4 (-9.8, 5.0) | | 0.53 |
| BMI^3^, kg/m^2^ | 0.6  (-0.7, 2) | | 0.37 | 0.3  (-1.1, 1.8) | | 0.65 | 0.01 (-2.1, 2.1) | | 0.99 |
| Depressive features^3^ | -1.4  (-2.4, -0.4) | | 0.01 | -1.38  (-2.2, 0.6) | | 0.001 | -2.2 (-3.9, -0.5) | | 0.02 |
| Back belief^3,4^ | 2.6  (0.4, 4.9) | | 0.02 | 2.2  (-0.1, 4.6) | | 0.06 | 3.8 (0.4, 7.3) | | 0.03 |
| **Change in score over 10 years** | | | | | | | | | |
| back belief score^3,5^ | 4.7  (2.3, 7.0) | | <0.001 | 4.7  (2.2, 7.2) | | <0.001 | 6.0 (2.4, 9.7) | | 0.001 |

^1^Data available for 415 participants who provided pain, disability and belief data at both time points.

^2^Data available for 417 for participants who provided pain and back belief, but not disability data at both time points.

^3^Data presented as Mean difference (95% confidence interval of the difference); comparison p-value for Independent T-test

^4^Lower back belief score indicates more negative back beliefs.

^5^Change in back beliefs was calculated by subtracting baseline (2006-2010) back belief score from follow-up (2016-2021), a negative value indicates developing more negative belief scores

**Supplementary Table S3**: **The difference in negative back belief scores between participants who did not develop and developed pain and/or disability, or between participants with no deterioration and deterioration of pain and/or disability from low-intensity symptoms**

| **Population defined at baseline by** |  | **No pain and/or disability at baseline** | |  | **Low pain and/or disability at baseline** | | |  |
| --- | --- | --- | --- | --- | --- | --- | --- | --- |
|  | Total (n)  Incident case (N) | No pain and or disability | Low/high pain and/or low/high- disability | P | Total (n)  Deteriorate (N) | No or low pain and/or disability | High pain and/or disability | P |
| **Pain and/or disability** | n=132  N= 71 | 31.1  (6.9) | 30.7  (5.8) | 0.71 | n= 283  N=29 | 30.5  (6.6) | 27.7  (6.4) | 0.03 |
| **Pain** | n= 138  N= 74 | 30.7  (7.3) | 30.8  (5.9) | 0.94 | n= 202  N=26 | 30.2  (6.4) | 27.6  (7.3) | 0.05 |
| **Disability** | n=402  N= 38 | 30.8  (6.5) | 27.8  (6.5) | 0.01 | n= 56  N= 10 | 24.92  (7) | 24.6  (4.9) | 0.91 |

Data presented as mean (standard deviation); comparison p-value for Independent T-test

A lower total score indicates negative back beliefs.

**Supplementary Table S4**: **The relationship between negative back belief scores and incident pain and/or disability, or between negative back belief scores and developing high-intensity pain and/or disability from low-intensity symptoms**

| **Population defined at baseline by** | **No pain and /or no disability at baseline** | | | **Low intensity-pain and/or low disability at baseline** | | |
| --- | --- | --- | --- | --- | --- | --- |
|  | **Total (n)**  **Incident case (N)** | **Unadjusted** | **Adjusted^1^** | **Total (n)**  **Deteriorate (N)** | **Unadjusted** | **Adjusted^1^** |
| **Pain and/or disability** | n=132  N= 71 | 1.01  (0.95-1.06) | 1.01  (0.95-1.06) | n= 283  N=29 | 1.06  (1.01-1.12) | 1.04  (0.98-1.10) |
| **Pain** | n= 138  N= 74 | 1.00  (0.94-1.05) | 1.00  (0.94-1.05) | n= 202  N=26 | 1.06  (1.0-1.12) | 1.03  (0.96-1.10) |
| **Disability** | n=402  N= 38 | 1.06  (1.02-1.12) | 1.06  (1.02-1.12) | n= 56  N= 10 | 0.99  (0.81-1.20) | 0.83 (0.57-1.22) |

Data were presented as odds ratio (95% Confidence Interval); examined using Binary Logistic Regression

^1^Adjusted for age, BMI, Low Mobility and completed secondary school or lower education level.

**Supplementary Table S5: Association of back belief and incidence of high-intensity pain/ high disability based on categories**

| Pain and disability | | P^1^ |
| --- | --- | --- |
| Depressive features (HADS score, 0-21) | | |
| <8  Persistent=365  Developed=32 | >8  Persistent=13  Developed=4 | 1 |
| 1.05  (1.00-1.11) | 1.05  (0.90-1.23) |  |
| BMI (median 27.2 kg/m^2^) | | |
| <27.2 kg/m^2^  Persistent=196  Developed=26 | >27.2 kg/m^2^  Persistent=177  Developed=10 | 0.6 |
| 1.08  (1.01-1.15) | 1.04  (0.96-1.15) |  |
| Age (median 60 years) | | |
| <60 years  Persistent=245  Developed=24 | >60 years  Persistent=134  Developed=12 | 0.4 |
| 1.04  (0.98-1.12) | 1.10  (1.01-1.19) |  |
| Mobility | | |
| Low mobility  Persistent=67  Developed=12 | High mobility  Persistent=312  Developed=24 | 0.3 |
| 1.11  (1.00-1.22) | 1.03  (0.97-1.10) |  |
| Education | | |
| Not completed secondary school  Persistent=148  Developed=15 | Completed secondary school  Persistent=224  Developed=21 | 0.1 |
| 1.01 (0.94-1.10) | 1.11 (1.03-1.19) |  |

Data were presented as odds ratio (95% Confidence Interval), and association was examined using Binary Logistic Regression (unadjusted)

^1^Interaction between groups.

**Supplementary Table S6: Association between negative back belief of each statement with developed high-intensity pain and/or high-disability at 10 years follow up**

| Back belief statements (proportion holding negative belief) | **Pain and/or disability**  **OR (95% CI)** | | **NNE** | **Pain**  **OR (95% CI)** | | **NNE** | **Disability**  **OR (95% CI)** | | **NNE** |
| --- | --- | --- | --- | --- | --- | --- | --- | --- | --- |
|  | Unadjusted | Adjusted^1^ |  | Unadjusted | Adjusted^1^ |  | Unadjusted | Adjusted^1^ |  |
| There is no real treatment for back pain (13%) | 1.09  (0.4, 2.93) | 0.95  (0.35, 2.62) | -160 | 0.68  (0.19, 2.31) | 0.57  (0.17, 1.99) | -17 | 1.87  (0.51, 6.55) | 1.68  (0.44, 6.42) | 13 |
| Back trouble will eventually stop you from working (18.3%) | 2.84  (1.36, 5.89) | 2.69  (1.23, 5.76) | 5 | 3.01  (1.40, 6.46) | 2.81  (1.27, 6.19) | 5 | 1.83  (0.56, 5.99) | 1.66  (0.49, 5.66) | 11 |
| Back trouble means periods of pain for the rest of one’s life (24.8%) | 1.58  (0.76, 3.29) | 1.54  (0.73, 3.24) | 11 | 1.21  (0.54, 2.71) | 1.16  (0.51, 2.62) | 35 | 1.72  (0.56, 5.25) | 1.66  (0.54, 5.13) | 9 |
| Back trouble makes everything in life worse (37.1%) | 1.58  (0.80, 3.14) | 1.51  (0.75, 3.02) | 12 | 1.56  (0.76, 3.22) | 1.50  (0.72, 3.12) | 10 | 2.33  (0.79, 6.84) | 2.23  (0.75, 6.59) | 5 |
| Back trouble may mean you end up in a wheelchair (9.4%) | 1.64  (0.60, 4.49) | 1.56  (0.56, 4.47) | 9 | 1.91  (0.69, 5.29) | 1.85  (0.65, 5.31) | 15 | 2.77  (0.74, 10.4) | 2.72  (0.70, 10.61) | 8 |
| Back troubles mean long period of time off from work (15.7%) | 1.92  (0.86, 4.30) | 1.80  (0.78, 4.14) | 18 | 2.30  (1.01, 5.23) | 2.12  (0.91, 4.92) | 8 | 2.23  (0.68, 7.34) | 2.19  (0.65, 7.44) | 8 |
| Once you have had back trouble there is always a weakness (41.4%) | 1.46  (0.74, 2.90) | 1.34  (0.66, 2.72) | 14 | 1.27  (0.62, 2.61) | 1.15  (0.54, 2.43) | 29 | 1.93  (0.66, 5.66) | 1.78  (0.59, 5.38) | 7 |
| Back trouble must be rested (27.0%) | 1.40  (0.67, 2.89) | 1.37  (0.65, 2.86) | 15 | 1.26  (0.58, 2.75) | 1.24  (0.56, 2.73) | 23 | 3.81  (1.29, 11.2) | 3.74  (1.26, 11.1) | 3 |
| Later in life back trouble gets progressively worse (36.4%) | 1.64  (0.82, 3.25) | 1.71  (0.85, 3.43) | 8 | 1.62  (0.78, 3.34) | 1.65  (0.78, 3.44) | 8 | 2.41  (0.82, 7.07) | 2.48  (0.83, 7.37) | 5 |

OR (95% CI), Odds ratio (95% confidence interval)

NNE Number needed to be exposed

^1^Adjusted for age, BMI, mobility (low mobility), and education (Completed secondary school or lower).
